# Antibodies in children with malaria to PfEMP1, RIFIN and SURFIN expressed at the *Plasmodium falciparum* parasitized red blood cell surface

**DOI:** 10.1038/s41598-018-21026-4

**Published:** 2018-02-19

**Authors:** Maria del Pilar Quintana, Jun-Hong Ch’ng, Kirsten Moll, Arash Zandian, Peter Nilsson, Zulkarnain Md Idris, Somporn Saiwaew, Ulrika Qundos, Mats Wahlgren

**Affiliations:** 1 Department of Microbiology, Tumor and Cell Biology (MTC), Karolinska Institutet, Stockholm, Sweden; 2 Department of Microbiology and Immunology, National University of Singapore, Singapore, Singapore; 3 Affinity Proteomics, Science for Life Laboratory, School of Biotechnology, KTH-Royal Institutet of Technology, Stockholm, Sweden; 4 Department of Parasitology and Medical Entomology, Faculty of Medicine, Universiti Kebangsaan, Malaysia Medical Centre, Kuala Lumpur, Malaysia; 5 Department of Clinical Tropical Medicine, Faculty of Tropical Medicine, Mahidol University, Bangkok, Thailand

## Abstract

Naturally acquired antibodies to proteins expressed on the *Plasmodium falciparum* parasitized red blood cell (pRBC) surface steer the course of a malaria infection by reducing sequestration and stimulating phagocytosis of pRBC. Here we have studied a selection of proteins representing three different parasite gene families employing a well-characterized parasite with a severe malaria phenotype (FCR3S1.2). The presence of naturally acquired antibodies, impact on rosetting rate, surface reactivity and opsonization for phagocytosis in relation to different blood groups of the ABO system were assessed in a set of sera from children with mild or complicated malaria from an endemic area. We show that the naturally acquired immune responses, developed during malaria natural infection, have limited access to the pRBCs inside a blood group A rosette. The data also indicate that SURFIN_4.2_ may have a function at the pRBC surface, particularly during rosette formation, this role however needs to be further validated. Our results also indicate epitopes differentially recognized by rosette-disrupting antibodies on a peptide array. Antibodies towards parasite-derived proteins such as PfEMP1, RIFIN and SURFIN in combination with host factors, essentially the ABO blood group of a malaria patient, are suggested to determine the outcome of a malaria infection.

## Introduction

Despite ongoing eradication efforts and a marked decrease in the number of malaria cases over the last 15 years, malaria is still endemic in 91 countries with an estimated of 212 million malaria cases and 429000 deaths during 2016, with *P. falciparum* being the most prevalent parasite in the African continent and the main responsible for the deadly cases^1^. Malaria clinical symptoms occur when parasites invade and multiply inside the human red blood cells (RBCs) where they transport proteins to the RBC cytoplasm and plasma membrane. These proteins confer adhesive characteristics to the parasitized RBCs (pRBCs) allowing their sequestration in the microvasculature, a hallmark process in the pathogenesis of severe malaria^2^. Sequestration is believed to occur via two main mechanisms, cytoadhesion (binding of pRBCs to endothelial cells lining the vasculature) and rosetting (clustering of RBCs around pRBCs). The rosetting phenomenon varies between isolates and has been linked to the development of severe disease^3–6^ and host phenotypes known to reduce the parasite rosetting capacity (e.g. thalassemic RBCs, HbS containing RBCs, low levels of CR1 and blood group RBCs) confer protection against the development of severe disease^7–9^. The ABO blood group is also important for the rosetting phenomena, with rosetting being more prominent in blood group A (group A) than in blood group O (group O)^10–13^. Moreover, children with group A RBCs suffering from malaria, are more likely to succumb to severe disease than children having group O^14–17^. Rosettes formed in the presence of group A RBCs have also been suggested as a mechanism to evade immune recognition by impairing antibody accessibility to parasite proteins on the surface of the pRBCs^18^. Parasite derived surface proteins include *P. falciparum* Erythrocyte Membrane Protein 1 (PfEMP1), repetitive interspersed family (RIFIN) proteins, subtelomeric variable open reading frame (STEVOR) proteins, surface-associated interspersed gene family (SURFIN) proteins and possibly others. The three first proteins mediate rosetting^13,19–21^ with PfEMP1 being the most studied of the three. The PfEMP1 N-terminal head structure including the N-terminal Sequence (NTS) and a Duffy Binding like domain (DBL1) has been identified as ligand both for rosetting and cytoadhesion^22^.

Exposure to *P. falciparum* in endemic areas induces a slow and gradual development of age-dependent immunity to clinical malaria, evidenced as a decline in the prevalence of both complicated and mild clinical episodes^23^. Early experiments where IgG from clinically immune adults was transferred to children infected with malaria, inducing a reduction in parasitaemia and alleviation of the clinical symptoms^24^, indicated that the naturally acquired immunity to malaria is mostly dependent on the production of an array of protective antibodies. Previous studies indicate that PfEMP1 on the pRBC surface is the major target of the immune response^25–29^. However, independent studies have also suggested that anti-RIFIN antibodies are a dominant component of the overall response against the pRBC surface^30,31^. Recent work has also isolated human monoclonal antibodies (from individuals living in an African endemic area) that cross-react with different isolates and recognize RIFINs on the surface of pRBCs^32^. Additionally, studies on the SURFIN family have detected signs of positive selection on the SURFIN_4.2_ predicted extracellular segment^33,34^, suggesting that this region of the protein is likely to be under host immune pressure due to its exposure on the pRBC and the merozoite surface^35^.

In this study, sera collected from children suffering from mild or complicated malaria living in a hyperendemic area in Buea Cameroon was used. The presence of antibodies against three surface parasite-derived proteins (PfEMP1, RIFIN-A and SURFIN_4.2_) was tested as well as the ability of the sera samples to recognize the native proteins (exposed on the pRBC surface of a model rosetting parasite FCR3S1.2), to disrupt rosettes and to induce phagocytosis. Additionally specific epitopes on the three proteins were identified as possible candidates involved in the rosetting phenotype. These parameters were also contrasted between parasites grown in group O or group A RBCs.

## Results

### Study population

A cross-sectional study was conducted in a malaria hyperendemic area in Buea, Cameroon. Detailed description of the sample collection has been published elsewhere^36^. The samples used in the present study included children between 6 months and 14 years of age (n = 176) presenting at collection time with mild (n = 112) or complicated malaria (n = 64). The latter presentation covered various complicated malaria syndromes including severe anemia (n = 20), severe respiratory distress (n = 5), cerebral malaria (n = 5) and all the other complicated presentations (n = 34) according the WHO criteria requiring hospital admission^2^. Table 1 summarizes demographic and clinical parameters for the samples included, presented as a total as well as stratified by malaria clinical presentation (mild/complicated). To determine if any of these parameters affected the outcome of the malaria clinical presentation, a conditional regression model was used. The analysis indicated that lower hemoglobin levels, higher axillary temperature and having an episode of fever (at the time of presentation at the health facility) were risk factors to develop complicated disease (Table 1). Gender, age, blood group and splenomegaly were similarly distributed in both groups and none of these parameters seemed to be a risk factor for developing complicated disease. In general, the proportion of males was slightly higher (51%) with predominance of young individuals (0–5 years). Majority of the children had blood group O (54%) followed by group A (22%), group B (18%) and group AB (6%). Hemoglobin levels were in average low (8.6 ± 2.3 g/dL) particularly in children suffering from complicated malaria (7.3 ± 2.3 g/dL), fever episodes were highly prevalent both at collection time (69%) as well as during the 24 hours before attending the health facility (89%).

**Table 1:** Clinical characteristics of children with mild and complicated malaria.

Characteristics	All	Mild	Complicated^a^	OR (95% CI)	*P* ^b^
	*N* = 176	*n* = 112	*n* = 64		
Gender^c^					0.421
Male	88 (51)	58 (53)	30 (47)	1.00 (reference)	
Female	85 (49)	51 (47)	34 (53)	1.29 (0.69–2.39)	
Age, median (IQR), years	4 (2–6)	4 (2–6)	3 (1–7)	0.99 (0.91–1.10)	0.955
Age group, years					0.374
0–5	113 (64)	71 (63)	42 (66)	1.00 (reference)	
6–10	54 (31)	37 (33)	17 (27)	0.78 (0.39–1.55)	
11–15	9 (5)	4 (4)	5 (8)	2.11 (0.54–8.31)	
Blood group^d^					0.464
O	95 (54)	62 (56)	33 (52)	1.00 (reference)	
A	39 (22)	27 (24)	12 (19)	0.84 (0.38–1.86)	
B	31 (18)	16 (14)	15 (23)	1.76 (0.77–4.01)	
AB	10 (6)	6 (5)	4 (6)	1.25 (0.33–4.75)	
Hemoglobin level, mean ± SD, g/dL	8.6 ± 2.3	9.4 ± 1.6	7.3 ± 2.7	0.96 (0.94–0.97)	**<0.001**
Axillary temperature^e^, mean ± SD, °C	38.3 ± 1.1	38.0 ± 1.0	38.7 ± 1.1	1.76 (1.29–2.40)	**<0.001**
Fever at admision (>37.5 °C)					**0.038**
No	52 (31)	39 (36)	13 (21)	1.00 (reference)	
Yes	117 (69)	68 (64)	49 (79)	2.16 (1.04–4.47)	
Fever episode^f^					0.055
No	19 (11)	16 (15)	3 (5)	1.00 (reference)	
Yes	152 (89)	92 (85)	60 (95)	3.48 (0.97–12.45)	
Splenomegaly^g^					0.277
No	72 (48)	46 (45)	26 (54)	1.00 (reference)	
Yes	79 (52)	57 (55)	22 (46)	0.68 (0.34–1.36)	

Data are number or proportion (%) of patients, unless otherwise indicated. Boldface type indicates statistical significance. ^a^Complicated malaria syndrome; severe malaria (*n* = 20), severe respiratory syndrome (*n* = 5), cerebral malaria (*n* = 5), and complicated (*n* = 34). ^b^A conditional logistic regression model was used to calculate the prospective odds of developing complicated malaria. ^c^3 Missing values. ^d^1 Missing value. ^e^7 Missing values. ^f^Fever during the last 24 hours before presenting at the health facility. ^g^25 Missing values.

### Naturally acquired antibodies to PfEMP1 (NTS-DBL1α domain), RIFIN-A and SURFIN_4.2_

**Figure 1:**
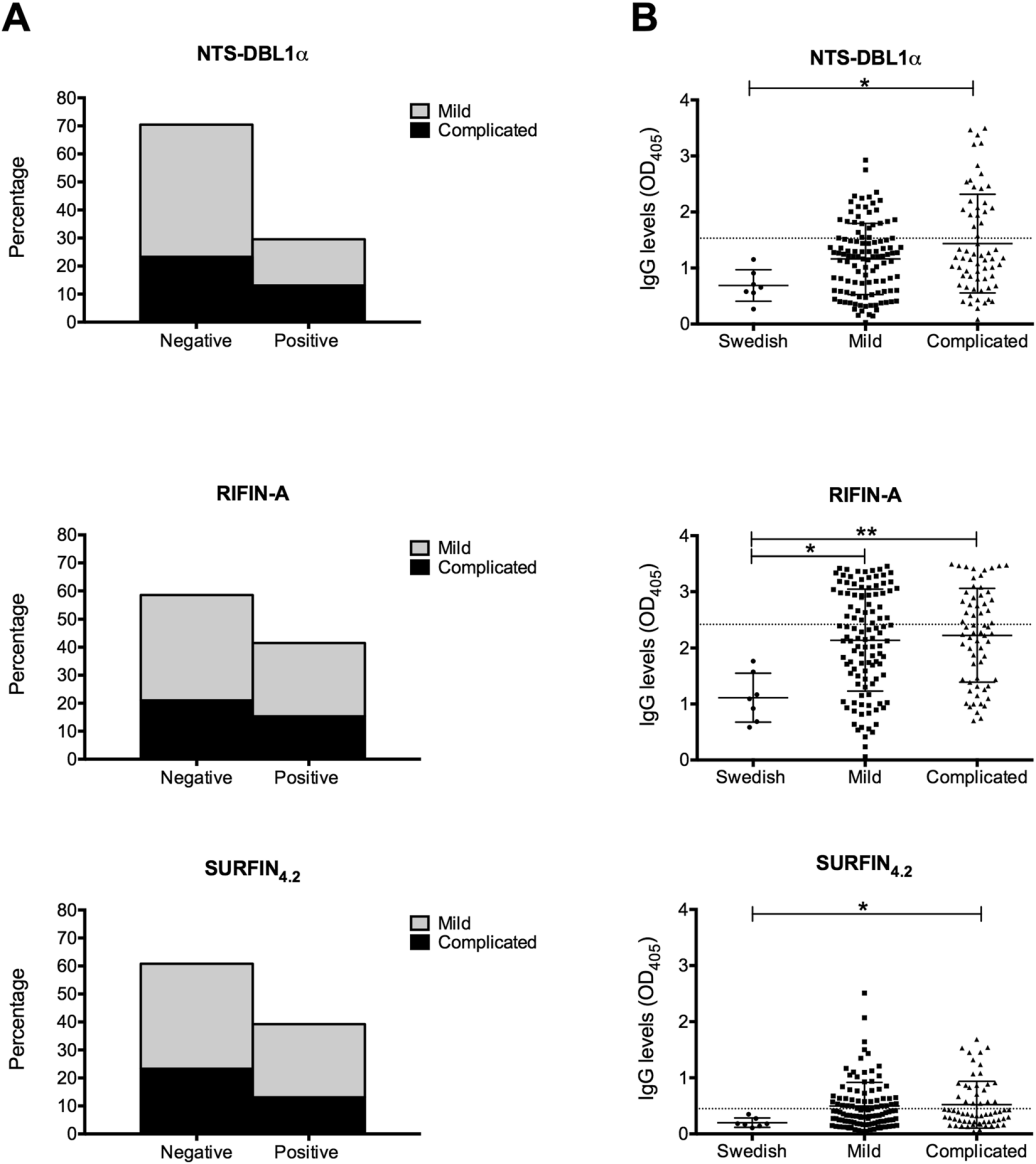
IgG levels against the NTS-DBL1α, RIFIN-A and SURFIN_4.2_. (**A**) Antibody seroprevalence in children with mild and complicated malaria against three surface proteins. (**B**) IgG levels compared between children with mild/complicated malaria and Swedish adults controls. Scatter dot plots show the means and SD. Differences between the groups were determined using a Kruskal-Wallis test. The dotted lines on each graph represent the threshold above which samples were considered as positive responders.

In order to determine whether antibodies towards three different surface proteins expressed by the model parasite FCR3S1.2 were present during natural infection with *P. falciparum*, recombinant proteins were expressed and used in ELISA. The NTS-DBL1α and the RIFIN-A used in this study have been clearly associated to the rosetting phenotype of this particular parasite strain^13,37,38^. The SURFIN_4.2_ is also expressed on the surface of this strain’s pRBCs, making it a potential mediator of rosetting/cytoadhesion as well as a target of naturally acquired immune responses^35^. Seroprevalence and antibody levels were measured in serum samples collected from children suffering from mild or complicated malaria living in Buea, Cameroon. These values were contrasted to the antibodies present in Swedish adults control sera from individuals that have not been previously exposed to malaria. As observed in Fig. 1A the seroprevalence of antibodies against the three proteins was between 29–41% among all the samples, demonstrating the presence of naturally acquired responses upon *P. falciparum* infection against the three proteins. However the number of positive responders was not associated with the development of a particular malaria clinical presentation (Table 2).

**Table 2:** Association of the measured variables with malaria clinical outcome.

Response	n^a^	Adjusted OR (95% CI)	*P* ^b^
NTS-DBL1α	52	1.87 (0.90–3.88)	0.092
RIFIN-A	73	0.94 (0.48–1.81)	0.845
SURFIN4.2	69	0.79 (0.39–1.59)	0.507
Multiplets group O RBCs	28	0.41 (0.14–1.16)	0.093
Positive cells group O RBCs	134	1.05 (0.48–2.31)	0.894
Multiplets group A RBCs	6	1.32 (0.08–20.95)	0.844
Positive cells group A RBCs	18	0.93 (0.20–4.24)	0.927
Phagocytosis group O RBCs	146	1.08 (0.44–2.64)	0.865
Phagocytosis group A RBCs	29	8.34 (1.42–49.08)	**0**.**019**

^a^Number of responders. ^b^Each response was tested for association with severe malaria in a multiple logistic regression adjusted for hemoglobin level, axillary temperature, and fever at admission. Malaria clinical presentation (Mild and Complicated) was the dependent variable while each of the measured responses were the independent variables. Boldface type indicates statistical significance.

In the endemic study area over 25% of children with mild malaria and over 35% of those with complicated malaria were seropositive for NTS-DBL1α. For the RIFIN-A over 41% of children with mild malaria and over 42% of those with complicated malaria were seropositive. A similar trend was observed for the SURFIN_4.2_ but with a slightly lower seropositivity in the complicated group (over 35%) (Table S1). For the three proteins, children presenting with complicated malaria had slightly higher IgG levels than those presenting with mild malaria, but the difference was not statistically significant (Fig. 1B). These results again suggest the lack of association between antibody levels against the three surface proteins tested and a particular malaria clinical presentation. When IgG titers against the three proteins were compared, it was apparent the low titers for SURFIN_4.2_ as compared with the other two proteins (mean OD across all samples of 0.505), this however, was not due to poor binding of the protein to the plate (data not shown) but rather represented a feature of this particular protein. For several samples tested on the RIFIN-A protein, OD values were close to 3.5, indicating they were close to reach the dynamic range limit for the assay. We therefore consider that the interpretation regarding the importance or irrelevance of the antibodies against this particular antigen should be carefully interpreted.

### Surface reactivity and rosette disruption activity

In order to address if the antibodies against the three different parasite proteins measured by ELISA, are likely to be of clinical importance, their surface reactivity (measured as percentage of IgG positive pRBCs) and their capacity to reduce the rosetting rate (measured by flow cytometry as percentage of multiplets) was measured.

All the 176 samples were tested on parasites grown in group O RBCs while only 49 (group A or group AB individuals that did not induce agglutination) were tested on parasites grown in group A RBCs. Out of the 176 samples used, 112 (64%) came from children with mild malaria while 64 (36%) corresponded to complicated malaria. For those tested on parasites grown in group A RBCs, 33 (67%) corresponded to mild malaria while 16 (33%) were from complicated malaria cases.

**Figure 2:**
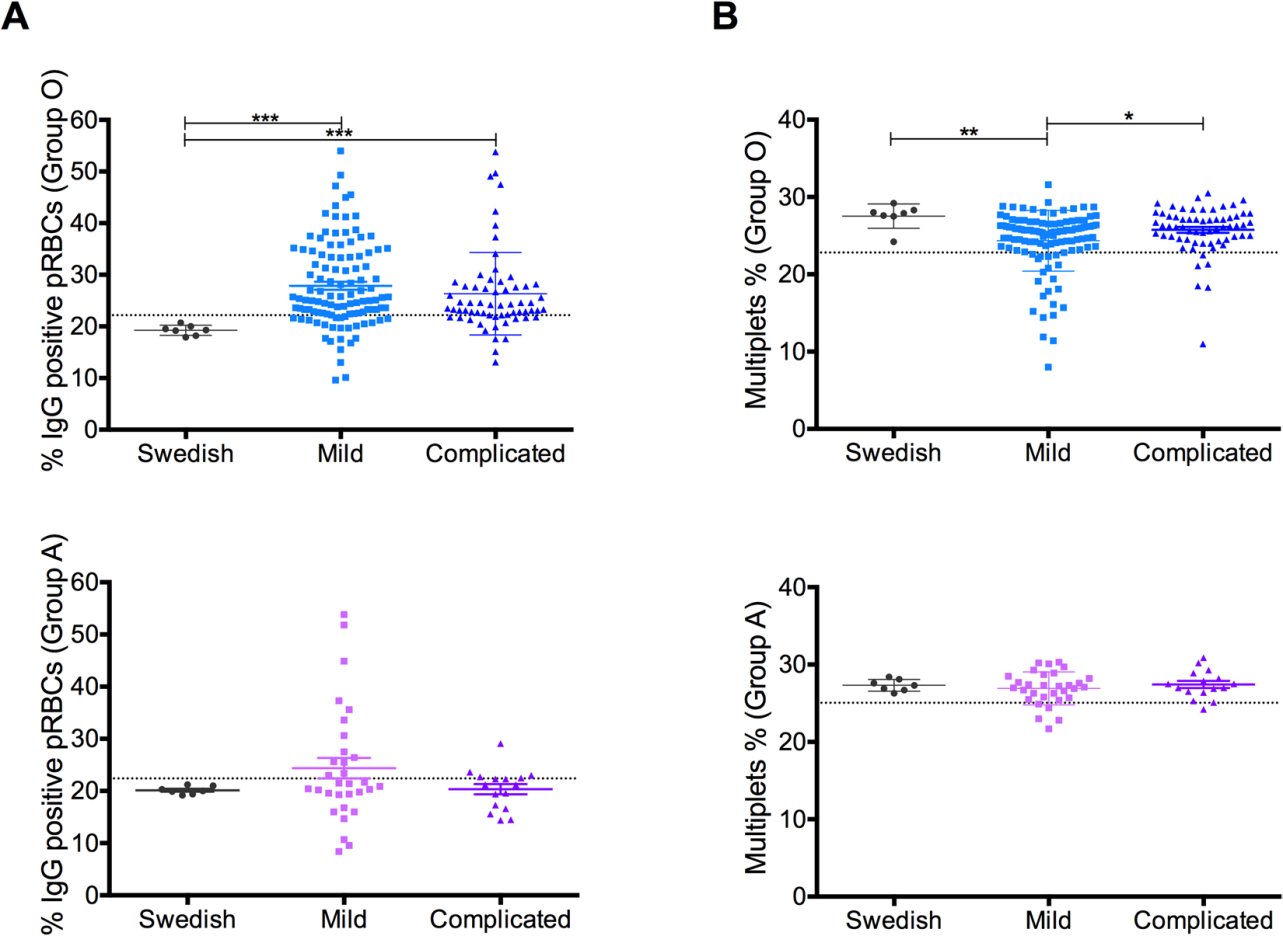
Surface reactivity and rosette disruption capacity. (**A**) Percentage of IgG positive pRBCs (measure of the surface reactivity) and (**B)** Percentage of multiplets (measure of the rosetting rate) in the presence of pediatric sera, both in group O (top panels) and group A RBCs (low panels) stratified by malaria clinical presentation (mild and complicated) and compared with Swedish adults controls. Scatter dot plots show the means and SD. Differences between the groups were determined using a Kruskal-Wallis test. The dotted lines on each graph represent the threshold above/below which samples were considered as positive responders.

Surface reactive antibodies were detected in 77% and 38% of the samples when tested in parasites grown in group O and group A RBCs respectively (Fig. 4A), indicating that if the serum was tested on parasites grown in group O RBCs the likelihood of them being reactive with the pRBC surface was around two times higher than when tested on parasites grown in group A RBCs. This observation suggests that the pRBC surface within a group O rosette is more accessible to the antibodies present in the sera tested as compared to group A rosettes. When the samples were stratified by malaria clinical presentation, 77% of the mild samples and 76% of the complicated samples were positive when tested on parasites grown in group O RBCs (Table S1). The average percentage of IgG positive pRBCs was similar between samples belonging to the two different clinical presentations when the samples were tested on parasites grown in group O RBCs, but significantly higher than the values generated by the Swedish adults controls (Fig. 2A upper panel), indicating that surface reactivity does not seem to be correlated with protection against complicated disease. When the samples were tested on parasites grown in group A RBCs, 42% of the mild samples and 31% of the complicated samples were positive (Table S1). Again there was no significant difference when the average percentage of IgG positive cells between samples belonging to mild or the complicated category were compared, and more importantly, there were no even significant differences when compared to the Swedish adult controls (Fig. 2A lower panel).

When the association between surface reactivity and the IgG levels against the three proteins was assessed, only a low positive correlation (r = 0.223, p = 0.003) was observed with anti SURFIN_4.2_ IgG levels (Fig. S1) and only when rosetting rate was measured on parasites grown in group O RBCs indicating a low association between the IgG titers against the three proteins and the surface reactivity measured.

To determine the levels of rosetting both in group O and group A, the percentage of multiplets was measured as described before^39^. Rosette disruption capacity was modest, being detected in 16% and 13% of the samples when tested on parasites grown in group O and group A RBCs respectively (Fig. 4A) and the rosetting rate was as likely to decrease in the presence of pediatric sera irrespectively of the blood group where the parasites tested were grown in. When the samples were stratified by malaria clinical presentation, 20% of the mild samples and 9% of the complicated samples were positive when tested on parasites grown in group O RBCs (Table S1). The average percentage of multiplets was slightly lower in the presence of pediatric sera from children suffering from mild malaria as compared with those with complicated malaria (24% vs. 26%) and this average was also lower compared with the Swedish adult controls (24% vs. 28%). In the presence of pediatric sera from children suffering from complicated malaria, the percentage of multiplets was similar to the one in the presence of Swedish adult control sera (26% vs. 28%) (Fig. 2B upper panel). When the samples were tested on parasites grown in group A RBCs, 16% of the mild samples and 6% of the complicated samples were positive (Table S1). There was no significant difference in the percentage of multiplets when samples belonging to mild, complicated or Swedish controls were compared, (Fig. 2B lower panel), in contrast to the difference observed when the samples were tested on parasites grown in group O RBCs.

When the association between surface reactivity and rosetting rate was assessed, a low negative correlation (r = −0.299, p < 0.0001 for group O and r = −0.350, p = 0.017 for group A) was observed between the two variables (Fig. S2), irrespectively of the blood group used to grow the parasites tested, with increasing levels of surface reactivity associated with lower rosetting rate.

When association between rosetting rate and the IgG levels against the three proteins was assessed, only low negative (r = −0.157, p = 0.037 and r = −0.324, p < 0.0001) correlations were observed with anti NTS-DBL1 and SURFIN_4.2_ IgG levels respectively (Fig. S3) when rosetting rate was measured on parasites grown in group O RBCs; suggesting an association between IgG levels for these antigens and the rosetting rate, with increasing levels of IgG generating lower levels of rosetting.

### Opsonization for phagocytosis

**Figure 3:**
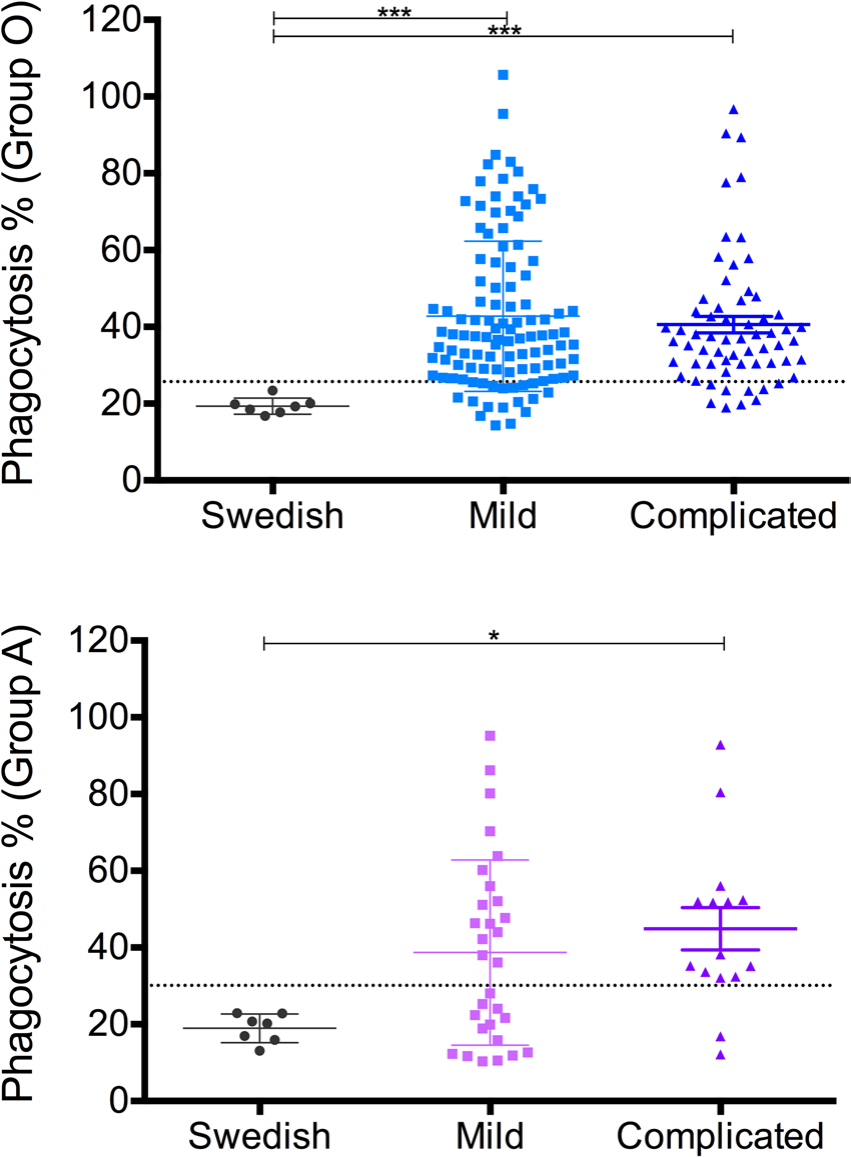
Opsonization for phagocytosis. Percentage of phagocytosis after opsonization of pRBCs -grown in group O (top panel) and group A RBCs (low panel)- with pediatric sera stratified by malaria clinical presentation (mild and complicated) and compared with Swedish adults controls. Scatter dot plots show the means and SD. Differences between the groups were determined using a Kruskal-Wallis test. The dotted lines on each graph represent the threshold above which samples were considered as positive responders.

**Figure 4:**
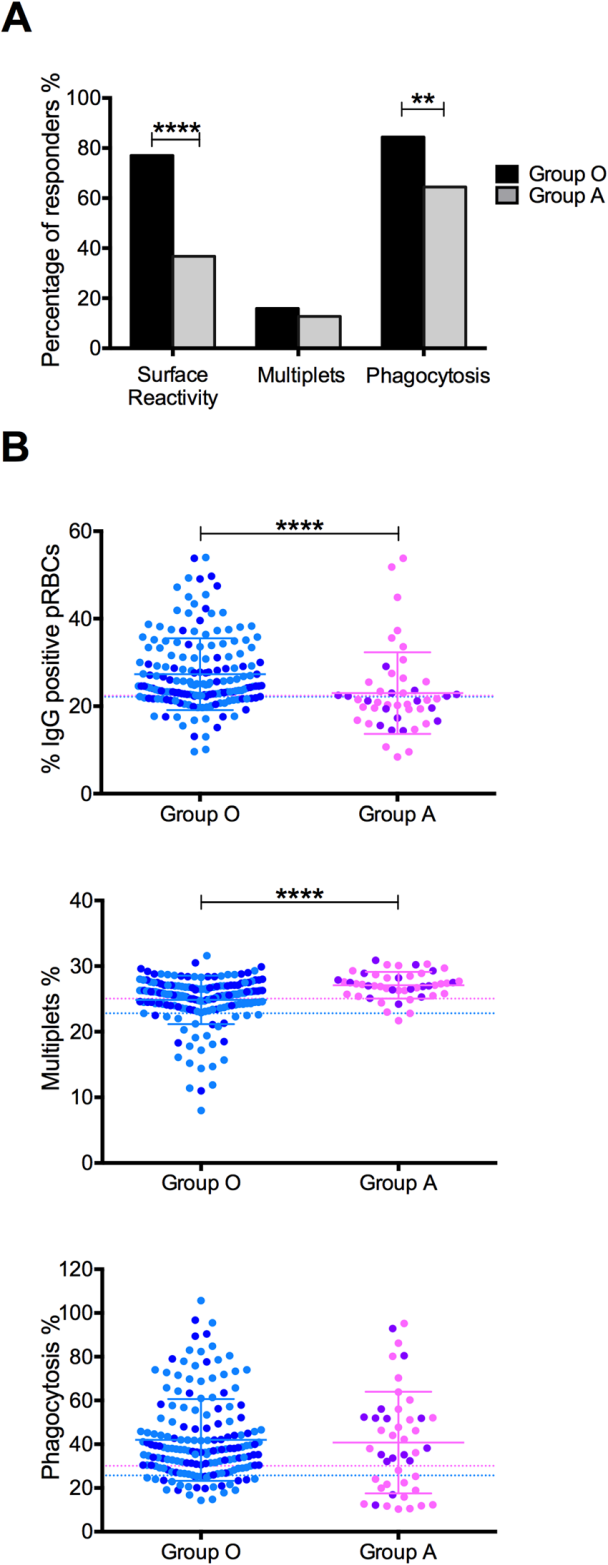
Comparison between the surface reactivity (measured as percentage of IgG positive pRBCs), the rosetting rate (measured as percentage of multiplets) and the percentage of phagocytosis in the presence of pediatric sera when the pRBCs tested were grown in group O vs. group A RBCs. (**A**) The percentage of positive responders for each variable is depicted. Differences between the percentages for each group were determined using a chi-square test. (**B**) Measurements for each of the variables are represented and grouped by blood group used to grow the parasites tested. Differences between the two groups were determined using a Mann-Whitney unpaired test.

To determine the opsonizing effect of pediatric sera *in vitro*, phagocytosis of pRBCs by THP-1 cells was measured as described previously^40,41^. Opsonization and induction of phagocytosis was prominent, being detected in 84% and 64% of the samples when tested on parasites grown in group O and group A RBCs respectively (Fig. 4A), indicating that if the sera were tested on parasites grown in group O the likelihood of them being phagocytized by THP-1 cells was around one and a half times higher than when tested on parasites grown in group A RBCs. When the samples were stratified by malaria clinical presentation, 84% of the mild samples and 86% of the complicated samples were positive when tested on parasites grown in group O RBCs (Table S1). The average percentage of phagocytosis was slightly higher in the presence of sera from children suffering from mild malaria compared with those with complicated malaria but this difference was not significant (43% vs. 41%) and this average was also considerably higher than the one generated by the Swedish adult controls (43% vs. 19%). In the presence of pediatric sera from children suffering from complicated malaria, the percentage of phagocytosis was also considerably higher than the one in the presence of Swedish adult controls (41% vs. 19%) (Fig. 3 upper panel). When the samples were tested on parasites grown in group A RBCs, 53% of the mild samples and 87% of the complicated samples were positive (Table S1), indicating an association between the percentage of phagocytosis and the clinical presentation of the serum donor, with sera from complicated cases being more likely to opsonize and induce phagocytosis when the parasites tested were grown in group A RBCs (Table 2). When the average percentage of phagocytosis was compared between samples belonging to mild, complicated or Swedish categories, significant difference was only found between the complicated and the Swedish adult controls (Fig. 3 lower panel) but not when compared to the samples belonging to the mild category.

When association between surface reactivity and percentage of phagocytosis was assessed, only a low positive correlation (r = 0.302, p < 0.0001) was observed between the two variables when samples were tested in group O grown parasites (Fig. S4) with increasing levels of surface reactivity associated with higher percentage of phagocytosis, indicating a low association between the percentage of phagocytosis and the surface reactivity.

When association between percentage of phagocytosis and the IgG levels against the three proteins was assessed, only a negligible and low positive correlation was observed with IgG levels against the three proteins (Fig. S5) when percentage of phagocytosis was measured on parasites grown in group O RBCs. When the same set of data was analyzed using 2 × 2 contingency tables, no statistical significance was found, reaffirming the low association between the IgG titers against the three proteins and the percentage of phagocytosis induced after pRBC opsonization.

### Comparison between variables measured on parasites grown in group O vs. group A RBCs

When the three variables measured in the presence of pediatric sera (surface, reactivity and opsonization for phagocytosis) were compared based solely on the percentage of positive responders, the group O set showed a larger percentage of positive responders compared to group A (Fig. 4A). Moreover if the average percentages were compared between group O and group A grown parasites (Fig. 4B), group O pRBCs seemed to be more accessible to the antibodies present in the sera tested (higher percentage of IgG positive cells) and were more sensitive to rosette disrupting antibodies present in the sera (lower percentage of multiplets). Significant difference was not observed when the percentage of phagocytosis was compared. These findings corroborate published findings showing a decreased accessibility to the surface of pRBCs embedded within a group A rosette.

To establish which of the variables measured in the study were determining the ability of the pediatric sera to perform a functional activity (disrupt the rosettes and opsonize the pRBC for phagocytosis), multiple linear regression models were used. Both rosette disruption and opsonization for phagocytosis (separately for group O and group A grown parasites) were treated as response variables, while the rest of the variables were considered predictors (antibody levels, surface reactivity for the corresponding blood group, age, hemoglobin levels and axillary temperature). Not surprisingly, the models (see Supplementary Analysis 1) indicated that both the capacity to disrupt the rosettes and to induce phagocytosis when the parasites tested where grown in group O RBCs (Models 1 and 2 in Supplementary Analysis 1) were related with the presence of surface reacting antibodies. Only IgG titers against SURFIN_4.2_ were on a borderline significant trend (p = 0.054) as predictor of the ability to reduce rosetting. Models for variables measured when the parasites were grown in group A RBCs were not statistically significant explained by the predictor variables included (Models 3 and 4 in Supplementary Analysis 1) and this again could reflect the inability of the antibodies present in sera to reach the pRBCs surface within a group A rosette.

### Reactivity on a surface antigen peptide array

**Figure 5:**
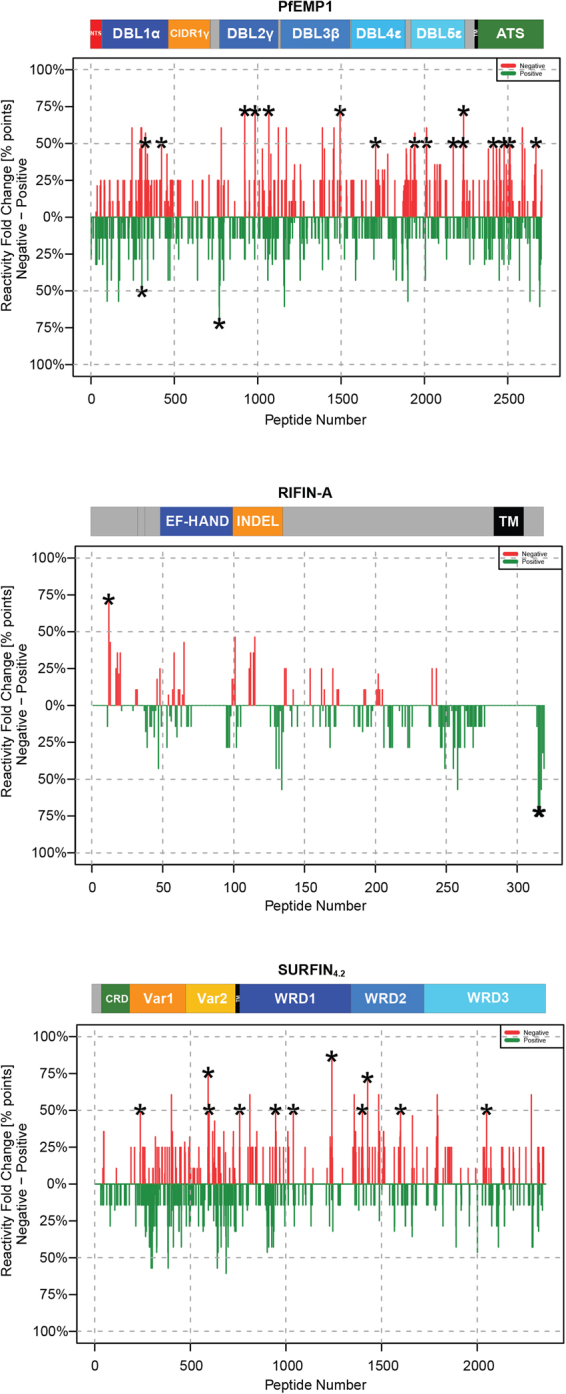
Pediatric sera reactivity against three surface proteins. A small subset of samples was selected and tested on a peptide array including three surface proteins. The samples analyzed were divided according to their ability to disrupt rosettes (of parasites grown in group O RBCs), with 7 being positive (in green) while 4 were considered negative (in red) for this assay. The stars indicate peptides differentially recognized between the two groups with an α ≤ 0.05.

To determine if the antibodies present in the sera tested, had particular specificities targeting particular regions of different parasite-derived surface proteins tested in this study that could also correlate with the sera ability to perform positively in the assays employed, a small set of samples was selected and tested on a peptide array encompassing the entire repertoire of several reported surface proteins families from 3D7 and IT4 parasites including the three proteins tested here. Samples were chosen based on clinical presentation and positivity/negativity for the various assays reported above (Table S2). When samples able to disrupt rosettes of parasites grown in group O RBCs were compared with those that did not, a few peptides differentially recognized were identified (Fig. 5 and Table S3). 19 epitopes were identified for PfEMP1, with 14 localized to the extracellular domain and 5 to the intracellular segment. Those localized to the extracellular domain were distributed along the sequence on the NTS-DBL1α (3 peptides), the DBL2γ (4 peptides), the DBL3β (1 peptide), the DBL4ε (1 peptide) and the DBL5ε (5 peptides) domains. For RIFIN-A only three peptides were identified, one to the extracellular domain and two to the intracellular fragment. 11 peptides were identified for SURFIN_4.2_, 3 localized to the extracellular segment, 1 to the TM domain and 7 to the intracellular domain. Epitopes localized on the extracellular domain were distributed on the first and the second variable regions (1 peptide on the Var1 region and 2 peptides on the Var2 region). Surprisingly most of the peptides identified (29 out of the 34) were more frequently recognized by samples that were not able to disrupt rosettes (Fig. 5, peaks in red) and only 3 were more frequently recognized by those samples with rosette-disrupting activity. However, the reported p values were not corrected for multiple testing, and should be regarded as indicative.

## Discussion

The rosetting phenotype varies among isolates and has been clearly associated to the progression of infected individuals into severe disease^3–6^, certain human phenotypes including the ABO blood group^7–9^ as well as the presence of rosette disrupting and opsonizing antibodies are considered a protective factor against severe disease development^42,43^. Studies in different endemic countries in Asia and Africa (reviewed by Cooling *et al*.^44^) have looked for associations between the ABO type and the risk to develop severe malaria in children, finding that group O individuals were protected. One study in particular found this protection to be accompanied by reduced rosetting rate, also finding a strong association between rosetting rate and severe malaria in A, B and AB individuals^9^. Rosetting has also been suggested as an evasion mechanism, where the surrounding RBCs work as “cloak” protecting the pRBCs from the host immune response^45^ with rosettes formed in group A RBCs being able to impair the recognition by rosette disrupting antibodies of the PfEMP1 on the surface^18^. In this study, sera from children naturally exposed to malaria and presenting with an episode of mild or complicated malaria were tested for their ability to recognize the pRBC surface of group O or group A grown parasites and more importantly for their ability to disrupt rosettes and opsonize for phagocytosis. Specific antibody targeting in three surface proteins (PfEMP1, RIFIN-A and SURFIN_4.2_) expressed by the model parasite FCR3S1.2 were also identified. Total IgG responses against recombinant PfEMP1, RIFIN-A and SURFIN_4.2_ were investigated. The proteins were selected based on their well-documented role in rosetting (PfEMP1 and RIFIN-A) and/or their localization on the pRBC surface (SURFIN_4.2_), which make them potential mediators in the development of complicated malaria as well as a target of naturally acquired immunity against clinical malaria. In addition different variables were also measured in the presence of these sera samples, namely, rosetting rate, surface reactivity and opsonization for phagocytosis on the rosetting parasite FCR3S1.2 grown in group O or group A RBCs, correlations of these variables and the total IgG responses against the three surface proteins were also assessed.

When IgG levels against the three proteins were measured, naturally acquired responses against the three proteins were observed, with seroprevalence among the samples ranging from 29% to 41% demonstrating that individuals exposed to natural infection develop antibodies that are able to recognize surface proteins expressed by an heterologous parasite (not the specific parasite causing the malaria episode). This indicates certain level of cross-reactivity despite the variability of the tested proteins, this has also been observed during controlled infections in naïve volunteers both for PfEMP1 and RIFIN^46^. When the possibility of differential seroprevalence between samples belonging to the mild or the complicated group was assessed, no significant association was found. This was surprising since previous studies have suggested that low antibody responses against variant surface proteins are associated with severe malaria^47^. However, it is possible that in this case, total IgG responses give inconsistent association with protection against severe disease. Previous studies focusing on merozoite proteins as well as on targets and mechanisms associated with protection, have shown that antibody affinity and biological efficacy are better predictors of protection (both against clinical and complicated malaria) than simple ELISA titers^48,49^. When prevalence for the other variables measured (surface reactivity, percentage of multiplets and opsonization for phagocytosis) was contrasted between mild and complicated samples, no association was found. This was surprising since it is widely accepted that the presence of antibodies against the pRBC surface would both prevent sequestration and opsonize pRBCs to induce phagocytosis therefore protecting the host from complicated disease. Also when the actual average values for each variable were compared between the two groups, only the percentage of group O multiplets showed a decrease after incubation with sera belonging to the mild malaria group. This suggests that sera from children suffering from mild malaria have antibodies that are able to disrupt slightly more efficiently rosettes formed in group O RBCs. However since the difference between the percentage of multiplets is only around 1.4% (which translates in an approximate rosette disruption capacity of only 5%), it is difficult to say if this could have any impact on the clinical outcome. It is also important to mention, that due to the cross-sectional nature of the study, the collected data represents only a snapshot of the patients therefore making it difficult to establish clear cause-effect relationships.

While the prevalence of antibodies against the pRBC surface was relatively high (77% and 38% when tested on parasites grown in group O and group A respectively), the presence of rosette disrupting capacity was in comparison relatively low (16% and 13% when tested on parasites grown in group O and group A respectively), indicating that there are many other targets on the surface than those involved in the rosetting phenomenon. This explanation is supported by the low correlation between the percentage of multiplets and the surface reactivity measured (Fig. S2). It also suggests that antibodies able to disrupt rosettes are not so frequently observed, as suggested in a previous study^50^ and that the antibodies present in the sera cross-react poorly with the PfEMP1 rosetting epitopes of the variant (IT4var60) expressed by the model parasite despite the good reactivity measured by ELISA. This is supported by other studies showing good cross-reactivity against PfEMP1 at the ELISA level but poor or null cross-reactivity with the native protein expressed on the pRBC surface^38,51^ and also by the results presented here, where neither the surface reactivity nor the percentage of multiplets were highly correlated to the IgG levels measured by ELISA against the particular PfEMP1 variant expressed by the model parasite FCR3S1.2 (Figs S1 and S2). Surprisingly, significant correlation between surface reactivity and the percentage of multiplets with the IgG levels was only found with SURFIN_4.2_. This suggests a potential role of this protein in rosetting, either as a direct ligand for RBC binding or as an accessory element for rosette formation.

The presence of opsonizing antibodies able to induce phagocytosis was relatively high (84% and 64% when tested on parasites grown in group O and group A respectively) in contrast to the anti rosetting activity, suggesting that there are other targets on the surface than those involved in the rosetting phenomena. However, the discrepancy in relation to the surface reactivity prevalence (77% and 38% when tested on parasites grown in group O and group A respectively) was puzzling, particularly when the samples were tested on parasites grown in group A RBCs. This was also observed through the low correlation between the surface reactivity and the percentage of phagocytosis only when samples were tested on parasites grown in group O while no correlation at all was found when tested on parasites grown in group A (Fig. S4). It is difficult to assess the reasons underlying the discrepancies between the two assays, since one would expect a strong correlation between surface reactivity and opsonization for phagocytosis. One way to address this would be to measure the IgG isotypes of the surface reacting antibodies; in particular of the IgG1 and IgG3 isotypes which are expected to interact with the Fc-receptors expressed by the THP-1 cells (CD64 and CD32) to trigger phagocytosis. Though we were constraint by the quantity of sera and could not perform these investigations, it is possible that a subset of antibodies with cytophilic activity (IgG1 and IgG3) are able to recognize equally pRBCs regardless the blood group of the rosette they are part of, therefore inducing similarly high percentages of phagocytosis despite different total surface reactivity. When different variables were compared between samples tested on parasites grown in group O versus those grown in group A RBCs, important differences were observed. In general any significant difference observed between the Swedish controls and the malaria patients’ sera was lost once the samples were tested on parasites grown in group A RBCs, (e.g. percentage of IgG positive pRBCs, percentage of multiplets Fig. 2 lower panels, and percentage of phagocytosis Fig. 3 lower panel). More importantly, both seroprevalence and average values for the different variables were different when measured on parasites grown in group O versus group A (Fig. 4A and B), supporting previous findings showing an impaired accessibility to the pRBC surface inside a group A rosette^18^. In this study this is most likely not only limited to the recognition of PfEMP1, but also of other targets on the surface (e.g. RIFIN, STEVOR, SURFIN_4.2_ and possibly others).

Though only a limited number of samples could be assayed on the ultra-dense peptide array, a few potentially important epitopes were identified. The peptides identified for the PfEMP1 preferentially recognized by samples with rosette-disrupting ability were localized on the DBL1α and 2γ domains. The peptide recognized on the DBL1α was localized by the end of helix 6 and immediately upstream of the SD3-loop, region that has been previously described as the main target of rosette disrupting antibodies, generated after animal immunization with this particular domain^38^. Even though the NTS-DBL1α has been described as the main rosetting mediator, a recent study has shown that antibodies against other DBL domains can also disrupt rosettes^40^, it is therefore possible that the peptide on the DBL2γ presented here represents one of the specific regions targeted by such antibodies. RIFIN-A has been clearly implicated in the rosetting phenotype of the parasite used, however it is not known which particular regions of the protein are involved on RBC binding and which part of the protein is targeted by rosette disrupting antibodies^13^. The results presented here could not identify a particular region targeted by such antibodies in the extracellular domain. The only region identified was on the intracellular segment, region that is not accessible to antibodies and cannot be part of direct interactions with the RIFIN-A receptor on the RBCs during rosette formation. SURFIN_4.2_ has been described as a pRBC surface antigen, however little is known regarding its function. The fact that it is partially co-transported with PfEMP1 on its route to the surface suggests that it could have a role at this particular cellular location^35^. The results presented here showed this could be the case since all the variables measured (rosetting rate, surface reactivity and phagocytosis induction) were correlated with the antibody levels measured for this antigen. Similarly as with RIFIN-A the peptide array analysis could not identify particular peptides preferentially targeted by samples with rosette disrupting activity. It is possible that regions targeted by rosette-disrupting antibodies are largely conformational and therefore difficult to identify when only linear peptides are being assessed. Surprisingly, most of the identified peptides were preferentially recognized by samples that did not disrupt rosettes (Fig. 5 and Table S3, peptides in red). These peptides could represent immunogenic regions of the proteins that act as an immunological smokescreen, diverting the generated antibody response from other more important regions (e.g. those involved in rosetting). This has been suggested to be the case, especially if the region contains amino acid repeats with high content of glutamic acid^52,53^. Due to the absence of strong correlations between the antibody levels against the three surface proteins and the rosette disruption capacity and the limited number of samples tested, the peptides described here require further verification.

In summary the data presented here shows that the acquired immune response developed during natural infection, has limited access to the pRBCs inside a group A rosette where the RBCs act as a cloak for the pRBCs, impairing the antibodies’ ability to recognize targets on the surface and perform their effector function. The data presented here also suggests that SURFIN_4.2_ localized at the pRBC surface could be involved in rosette formation either as a direct ligand or as an accessory element for rosette strengthening.

## Methods

### Parasite cultures

The rosetting *P. falciparum* FCR3S1.2 strain was cultured according to standard methods. Red blood cells group O or A in the presence of A^+^ non-immune Swedish serum were used and the culture flasks were gassed with 90% NO_2_, 5% O_2_ and 5% CO_2_ and placed in a 37 °C shaker incubator. Parasites were routinely synchronized at ring stage by sorbitol treatment^54^ and the rosetting phenotype was maintained by enrichment over a Ficoll cushion as previously described^55^.

### Recombinant proteins

The PfEMP1 NTS-DBL1α-domain was expressed as previously described^51^. The DNA sequences encoding for the predicted extracellular domains for the RIFIN (PF3D7_0100400, sequence encoding amino acids 38 to 330) and the SURFIN (PFIT_0422600, sequence encoding amino acids 1 to 746) proteins were codon optimized and cloned into the pJ414express vector (DNA2.0) and the pDest527 (kind gift from Dominic Esposito, Addgene plasmid #11518) respectively. Proteins were expressed as a C-terminal (RIFIN) or N-terminal (SURFIN_4.2_) 6× histidine-tagged protein in *Escherichia coli* (BL21, New England Biolabs). Both proteins were solubilized from washed inclusion bodies (IB) with denaturing solution for 2 hours at room temperature. The proteins were refolded by the rapid dilution method, 25 mg of protein were reduced with 10 mM DTT for 1 hour at room temperature and the solution added drop wise into ice-cold refolding buffer. After refolding for ≈24 hours at 4 °C, the proteins were dialyzed against PBS and concentrated using Amicon Ultracel centrifugal filter units (Millipore). All proteins were purified over a Ni-NTA column (Qiagen) and eluted with imidazole. The purified proteins were analyzed by sodium dodecyl sulfate polyacrylamide gel electrophoresis (SDS-PAGE) obtaining a single band with the expected size (Fig. S6).

### Serum samples

Sera sample collection was done in Buea, Cameroon and it has been described before^36^. Collection was done between March and May of 2007 (rainy season), with an estimated entomological inoculation rate in this area of around 0.56 infected mosquito bites per person per night^56^. The study included children, positive for malaria infection by microscopy. Malaria clinical presentation was classified as mild or complicated. Mild malaria cases were defined as patients with a positive blood smear for *P. falciparum* without complicating manifestations and treated as outpatients. Complicated malaria cases were defined as patients requiring hospitalization and intravenous treatment with quinine or arthemether due to anemia, hyperparasitemia (parasitemia >5%), hyperpyrexia, seizures, prostration and/or vomiting. Sera samples were heat inactivated before being used in all the assays described in this study.

### ELISA

The presence of antibodies recognizing the three recombinant proteins were measured by ELISA (enzyme-linked immunosorbent assay) as previously described^57^. Recombinant proteins (5 μg, dissolved in 15 mM Na_2_CO_3_ and 35 mM NaHCO_3_, pH 9.6) were coated onto Maxisorp plates (Nunc, Rosklid). Blocking was performed with 1% bovine serum albumin (BSA) in PBST. Sere samples were incubated with the coated plates for one hour at room temperature at a 1:1000 dilution. Plates were thoroughly washed with PBST and IgG was detected with alkaline phosphatase-conjugated goat anti-human IgG (Sigma). Plates were again thoroughly washed and developed with SigmaFast p-nitrophenyl phosphate tablets (Sigma). Readout was optical density (OD) at 405 nm (Multiskan^TM^ GO Microplate Spetrophotometer, Thermo Scientific). A control pool of Swedish donors was included, as well as control wells without serum where PBS was used instead (background). Samples were considered seropositive when OD_405_ was higher than the mean plus three standard deviations of the Swedish control samples.

### pRBC surface reactivity

IgG binding to the pRBCs surface was detected by flow cytometry as previously described^42^. Briefly, 2% fetal bovine serum (FBS) in PBS was used to block pRBCs at trophozoite stage. pRBCs were then incubated with the human serum samples in a dilution 1:5 for one hour at 37 °C. After three thorough washes, IgG was detected with a secondary antibody goat anti-human IgG coupled to Alexa488 (Molecular Probes®, Life Technologies, dilution 1:200). To stain the parasite nuclei Hoechst at 10 μg/ml was added together with the secondary antibody during the last 30 min of the incubation. After a final thorough round of washes pRBCs were analyzed by flow cytometry. Control sera from Swedish donors that have never been exposed to malaria infection were included. Surface reactivity was expressed as percentage of IgG positive pRBCs. Samples were considered as positive if this percentage was higher than the non-exposed Swedish control samples average plus three standard deviations.

### Rosette disruption assay

The ability of pediatric sera samples to disrupt rosettes formed by the parasite used, was measured as previously described^39^. A control pool of six Swedish donors never exposed to malaria infection was included as well as a negative control where no serum was added. Samples were tested in a 1:5 dilution, incubated for 1 hour at room temperature. Parasite nuclei were stained with Hoechst-DHE (Invitrogen). Percentage of multiplets (events falling outside the 45° plane in the forward scatter area vs. forward scatter height plot) in the double stained Hoechst-DHE population was measured by flow cytometry and taken as a read out of rosetting rate. Samples were considered positive if percentage of multiplets was lower than the mean minus three standard deviations of the Swedish control samples.

### Phagocytosis assay

pRBC phagocytosis by THP-1 cells was measured as previously described^40,41^ using the human monocytic line THP-1. Synchronized and purified pRBCs were used, disrupting the rosettes mechanically using a 23G syringe. The pRBCs were stained in ethidium bromide solution and distributed in a rounded-bottom 96-well plate. Cells were centrifuged and the pellet was re-suspended in the tested serum sample (1:5 dilution). A positive control (anti human red blood cells, ab34858, ABCAM, 1:100 dilution) and a negative control (unopsonized control) were always included as well as sera control from Swedish individual without previous exposure to malaria infection. The opsonization was allow to occurs for 45 minutes at 37 °C for 45 minutes, after which, opsonized pRBCs were then added onto the THP-1 cells and co-incubated for 40 minutes at 37 °C, 5% CO_2_. Phagocytosis was blocked by centrifugation at 4 °C followed by a wash with ammonium chloride solution (15 mM NH_4_Cl, 10 mM NaHCO_3_, 1 mM EDTA) to remove non-phagocytized pRBCs. Excessive lysis was blocked by addition of PBS supplemented with 2% FBS, followed by three through washes. Cells were analyzed by flow cytometry (FACSCalibur, BD Biosciences) and the phagocytosis rate was calculated relative to the positive control. Samples were considered positive if relative phagocytosis was higher than the non-exposed Swedish control samples average plus three standard deviations.

### Peptide Array

Custom ultra-dense peptide microarrays obtained from Roche-Nimblegen were applied for epitope mapping as described before^58^. An array containing 175,000 peptides of 12 amino acids in length and with an 11-residue overlap was designed to include several reported *P. falciparum* surface proteins, including the 2TM family, PHISTs, RIFINs, STEVORs, SURFINs and a handful PfEMP1s as reported in plasmodb, in all cases sequences annotated as pseudo-genes were excluded. Duplicated (conserved) protein sequences were deleted to reduce the total number of peptides. A small set of pediatric sera was used and added to individual peptide arrays (Supplementary Table 2). Total IgG binding was detected using a secondary antibody (Alexa 647 conjugated anti-human IgG, cat. 709-606-149, Jackson Immunoresearch) and slide scanning at 2 µm resolution (MS200, Roche NimbleGen Inc., Madison, WI). Each spot on the array was subjected to pre-filtration criteria, as described previously^59^, to define reactivity and also minimizing false positives: by requiring the spot MFI to be above two times the local spot background MFI and maximum 50% coefficient of variation within the spot. The samples were divided according to their ability to decrease the percentage of multiplets (positive vs. negative) and the reactivity for each peptide was dichotomized as positive or negative for each sample to perform binary analysis using a Barnard’s test. Peptides differentially recognized by one group or the other were considered bona fide if p value was below 0.05. No correction for multiple testing was performed due to the low number of samples in the compared groups.

### Analysis

Flow cytometry analysis was performed using the FlowJo version 9.2 and 10.0.7 software (TreeStar, USA). Statistical analysis was performed using the GraphPad Prism version 6.0 f for Mac OS X (La Jolla, California, USA) and the statistical software package STATA/SE 13.1; for all the analysis an alpha level of 0.05 was used to determine statistical significance. In Figures where asterisks were used to represent statistical significance, the following convention was followed: p > 0.05 not significant, p ≤ 0.05*, p ≤ 0.01**, p ≤ 0.001*** and p ≤ 0.0001****. To determine if any of the population characteristics (gender, age, blood group, hemoglobin level, temperature, fever and splenomegaly, see Table 1) were potential predictors of the malaria clinical presentation (mild vs. complicated), logistic regression was used and only factors that showed evidence of a significant effect (p < 0.05) were used to adjust a multivariate logistic regression model where the variables measured in this study (antibody levels against the three surface antigens, surface reactivity, rosette disruption and opsonization for phagocytosis, see Table 2) were assessed for their association with the malaria clinical presentation. Differences in antibody levels, surface reactivity, rosette disruption capacity and opsonization for phagocytosis between Swedish control, children with mild malaria and complicated malaria were assessed using a Kruskal-Wallis test followed by a Dunn’s multiple comparison test. Spearman correlations were used to determine association between variables. To establish which characteristics were determining the ability of the pediatric sera to perform a functional activity (disrupt the rosettes and opsonize the pRBC for phagocytosis) multiple linear regression models were used, where both rosette disruption and opsonization for phagocytosis were considered as response variables, while the rest of the variables were considered predictors (antibody levels, surface reactivity for the corresponding blood group, age, hemoglobin levels and axillary temperature). VIF (variance inflation factor) calculation was performed to rule out co-linearity between the predictor variables.

### Ethics statement

Ethical permission for human sample collection was approved by the Ministry of Basic Education, Republic of Cameroon (ethical permit number: G379/900) and the Regional Ethical Review Board in Stockholm, Sweden (ethical permit number: 2006/1323-13/1), with informed consent obtained from the children parents/guardians. We confirm that all the methods were performed in accordance with the relevant guidelines and regulations.

## Electronic supplementary material

Supplementary Information
