# Supplementary material for: Antibodies in children with malaria to PfEMP1, RIFIN and SURFIN expressed at the Plasmodium falciparum parasitized red blood cell surface
Source: Sci Rep. 2018 Feb 19;8:3262. doi: 10.1038/s41598-018-21026-4 (PMC5818650; doi:10.1038/s41598-018-21026-4)
Supplement: Supplementary file 1 — Supplementary Information [file 41598_2018_21026_MOESM1_ESM.pdf]

Supplementary information

Antibodies in children with malaria to PfEMP1, RIFIN and SURFIN expressed at the *Plasmodium falciparum* parasitized red blood cell surface

Maria del Pilar Quintana[1*], Jun-Hong Ch'ng[1,2], Kirsten Moll[1], Arash Zandian[3], Peter Nilsson[3], Zulkarnain Md Idris[1,4], Somporn Saiwaew[5], Ulrika Qundos[3] & Mats Wahlgren[1*]

[1]Department of Microbiology, Tumor and Cell Biology (MTC), Karolinska Institutet, Stockholm, Sweden. [2]Department of Microbiology and Immunology, National University of Singapore, Singapore. [3]Affinity Proteomics, Science for Life Laboratory, School of Biotechnology, KTH-Royal Institutet of Technology, Stockholm, Sweden. [4]Department of Parasitology and Medical Entomology, Faculty of Medicine, Universiti Kebangsaan, Malaysia Medical Centre, Kuala Lumpur, Malaysia [5]Department of Clinical Tropical Medicine, Faculty of Tropical Medicine, Mahidol University, Bangkok, Thailand.

* Corresponding authors: mats.wahlgren@ki.se, pilar@sund.ku.dk

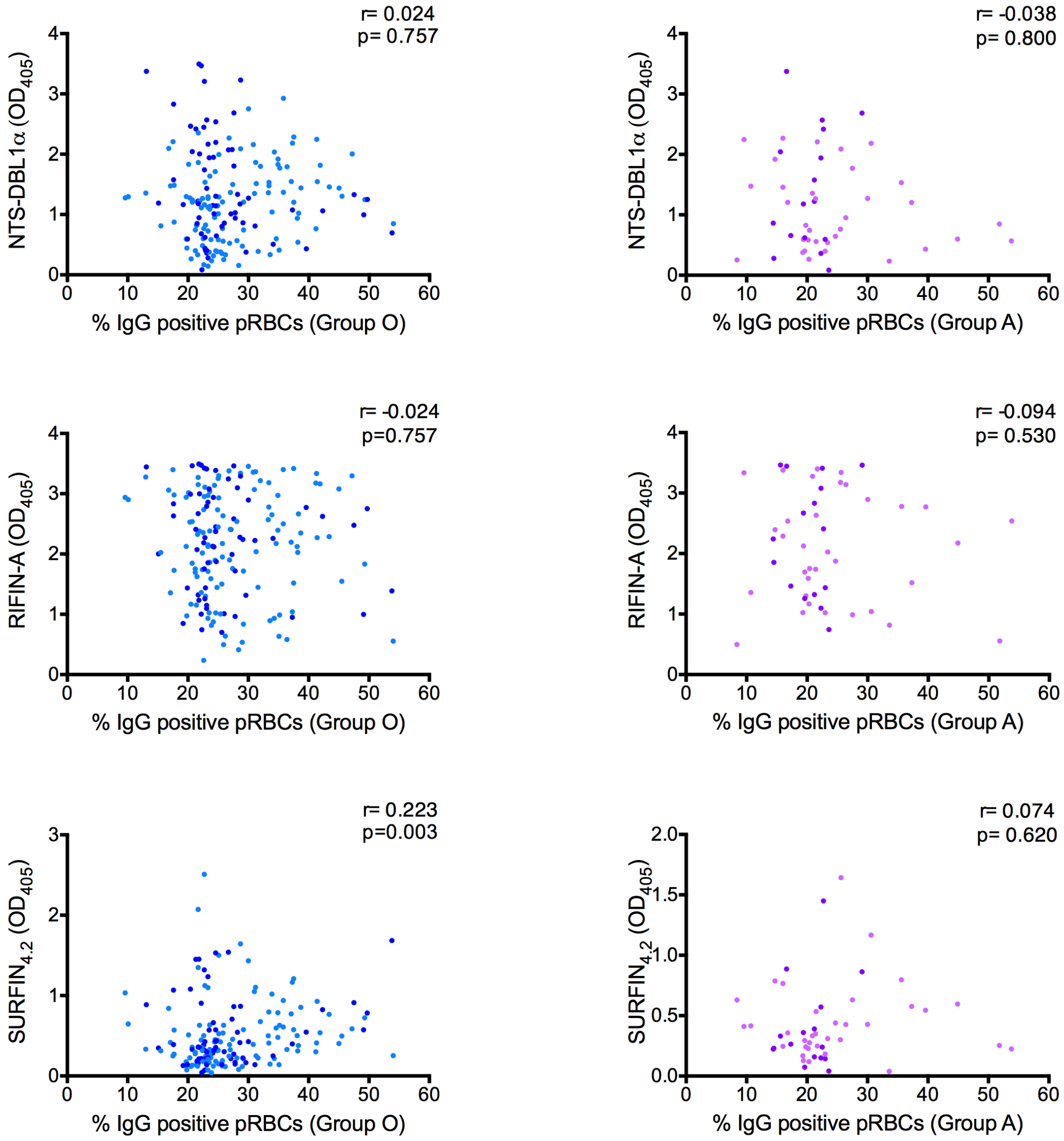

Fig. S1. Correlation between surface reactivity (measured as percentage of IgG positive pRBCs) and the IgG levels against the NTS-DBL1α, RIFIN-A and $SURFIN_{4.2}$ in children with mild and complicated malaria. Blue and purple dots represent samples tested on parasites grown in group O and group A RBCs respectively (darker shade of color represents samples classified as complicated malaria). Correlation is represented by a Spearman's rank correlation coefficient r.

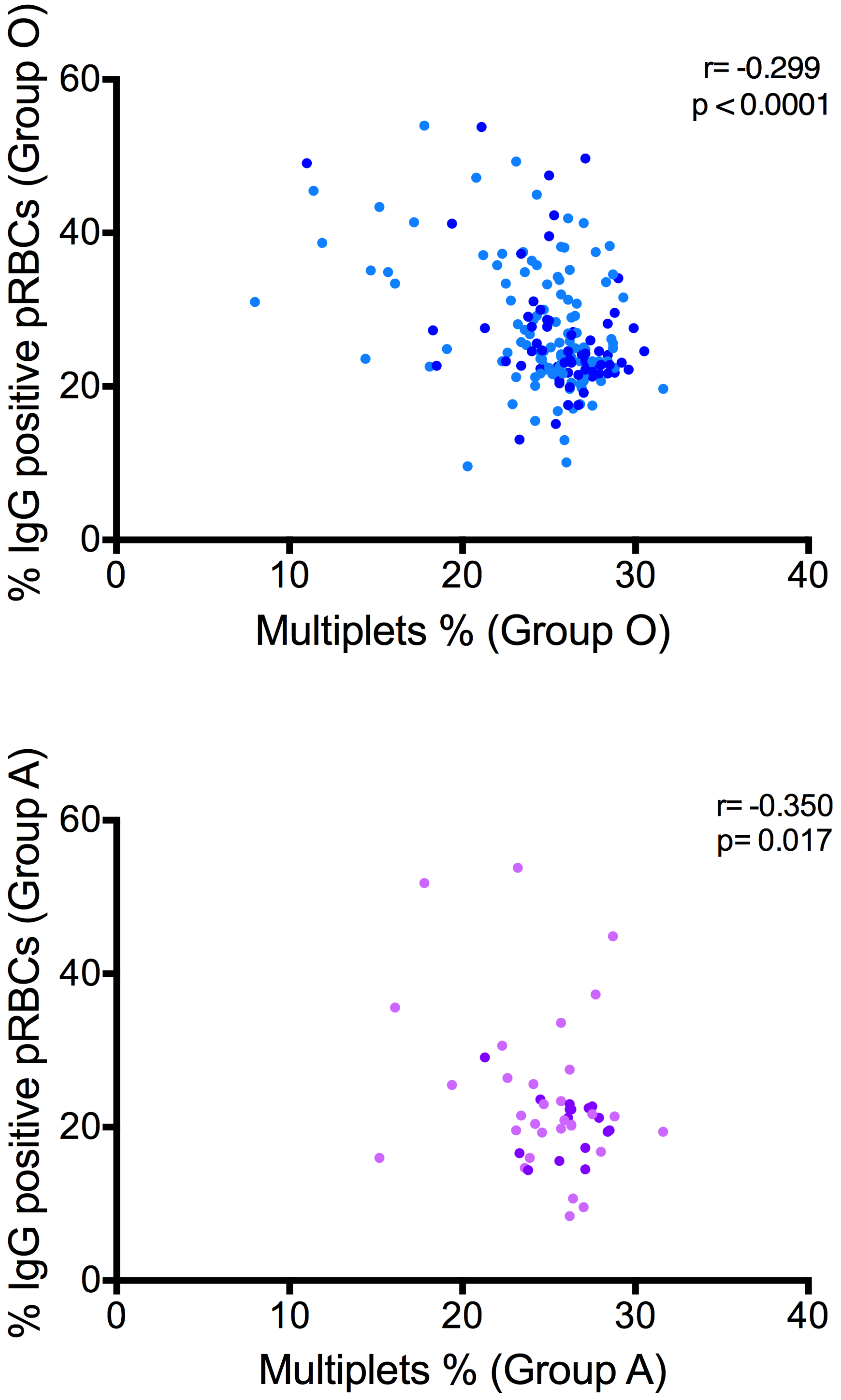

Fig S2. Correlation between rosetting rate (measured as percentage of multiplets) and the presence of naturally acquired antibodies (IgG) against the pRBC surface (measured as percentage of IgG positive cells). Blue and purple dots represent samples tested on parasites grown in in group O and group A RBCs respectively (darker shade of color represent samples classified as complicated malaria). Correlation is represented by a Spearman's rank correlation coefficient r.

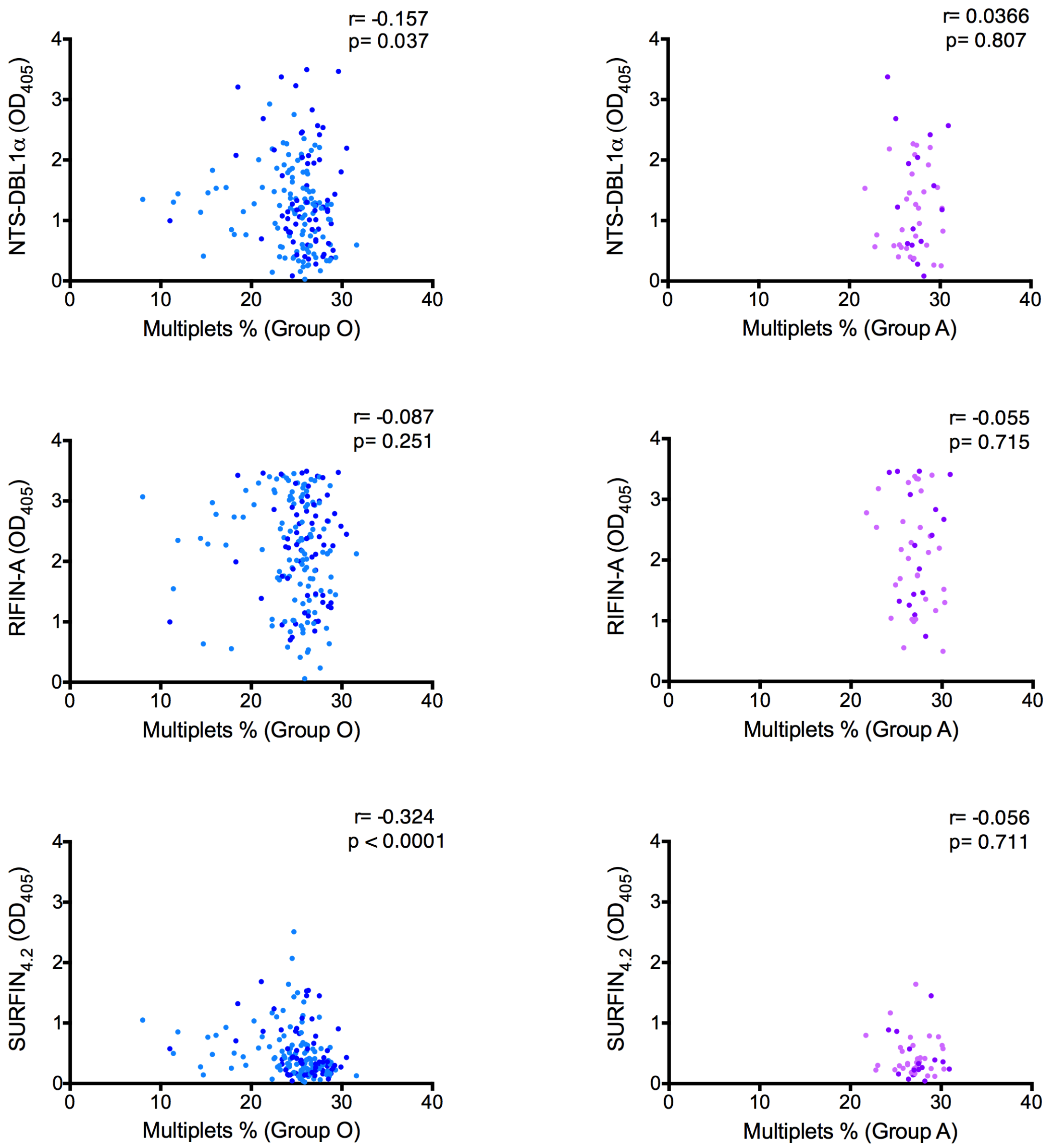

Fig. S3. Correlation between rosetting rate (measured as percentage of multiplets) and the IgG levels against the NTS-DBL1α, RIFIN-A and $SURFIN_{4.2}$ in children with mild and complicated malaria. Blue and purple dots represent samples tested on parasites grown in in group O and group A RBCs respectively (darker shade of color represents samples classified as complicated malaria). Correlation is represented by a Spearman's rank correlation coefficient r.

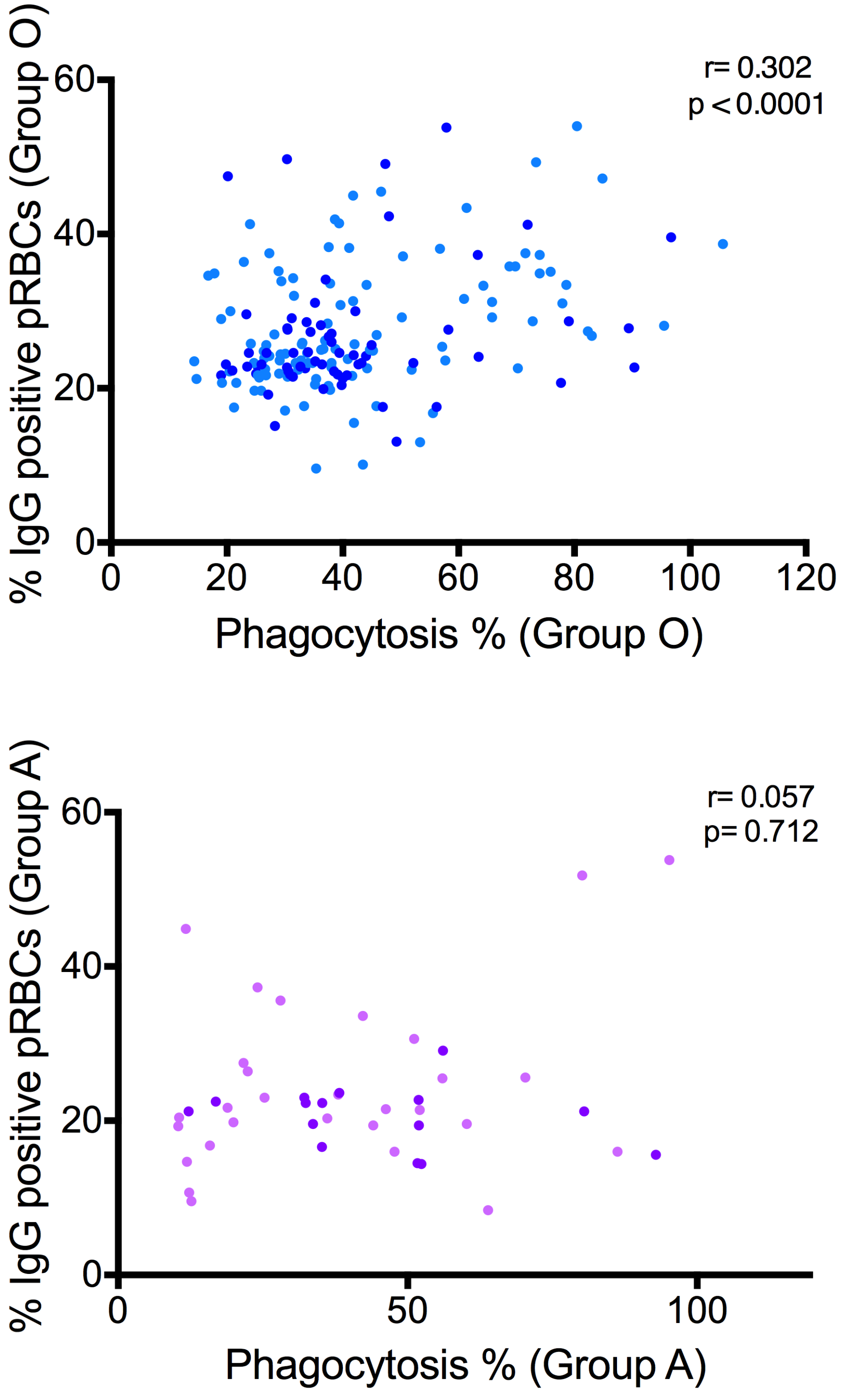

Fig S4. Correlation between percentage of phagocytosis and the presence of naturally acquired antibodies (IgG) against the pRBC surface (measured as percentage of IgG positive cells). Blue and purple dots represent samples tested on parasites grown in in group O and group A RBCs respectively (darker shade of color represent samples classified as complicated malaria). Correlation is represented by a Spearman's rank correlation coefficient r.

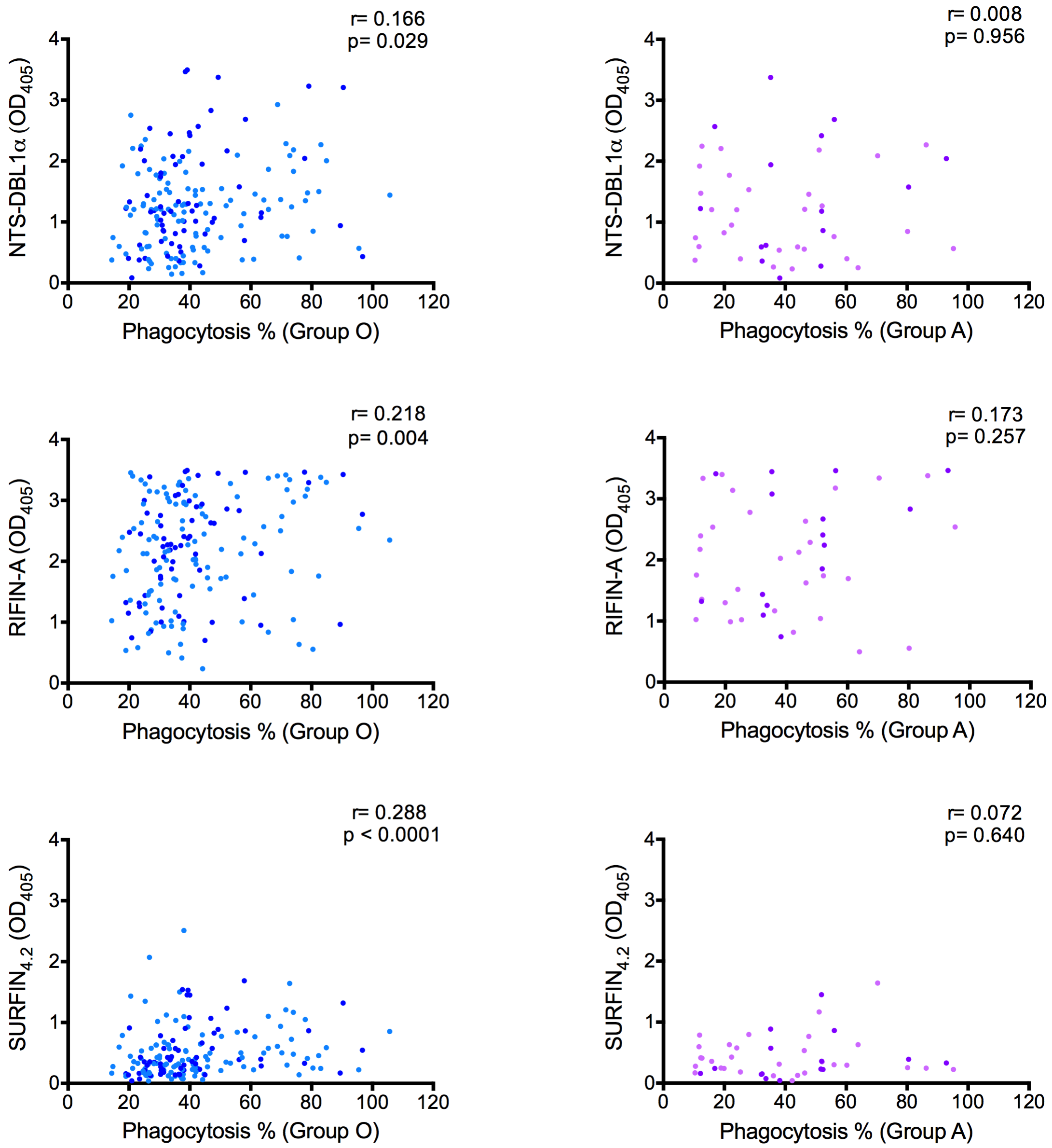

Fig. S5. Correlation between percentage of phagocytosis and the IgG levels against the NTS-DBL1α, RIFIN-A and $SURFIN_{4.2}$ in children with mild and complicated malaria. Blue and purple dots represent samples tested on parasites grown in in group O and group A RBCs respectively (darker shade of color represent samples classified as complicated malaria). Correlation is represented by a Spearman's rank correlation coefficient r.

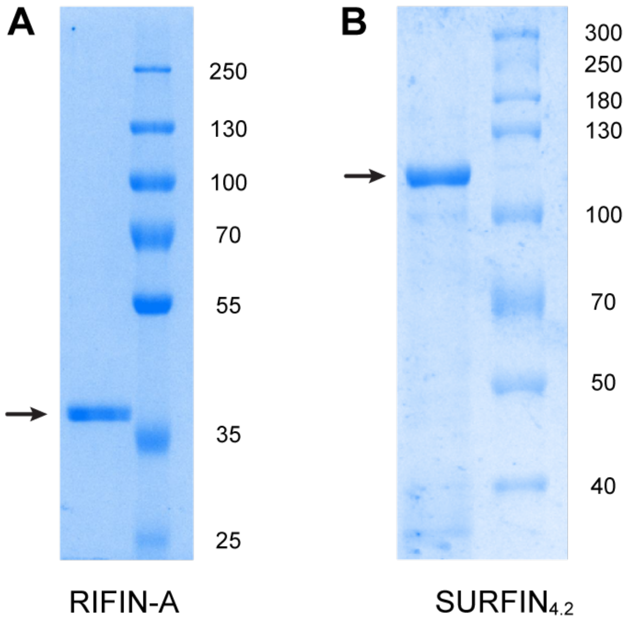

Fig. S6. Coomassie stained gel of recombinant proteins (RIFIN-A and $SURFIN_{4.2}$) expressed in *E. coli* and purified by IMAC.

		Percentage of responders						
		Mild		Complicated		Total		
		Positive	Negative	Positive	Negative	Positive	Negative	p-value**
ELISA	NTS-DBL1α	25.89	74.11	35.94	64.06	29.55	70.45	0.16
	RIFIN-A	41.07	58.93	42.19	57.81	41.48	58.52	0.8851
	$SURFIN_{4.2}$	41.07	58.93	35.94	64.06	39.20	60.80	0.5022
Surface Reactivity	Group O	77.48	22.52	76.19	23.81	77.01	22.99	0.8462
	Group A	41.94	58.06	31.25	68.75	38.30	61.70	0.4752
Percentage of multiplets	Group O	19.64	80.36	9.38	90.63	15.91	84.09	0.0732
	Group A	16.13	83.87	6.25	93.75	12.77	87.23	0.3362
Opsonization for phagocytosis	Group O	83.64	16.36	85.71	14.29	84.39	15.61	0.7171
	Group A	53.33	46.67	86.67	13.33	64.44	35.56	0.0277

Table S1. Prevalence of seropositivity for the different variables measured, stratified by malaria clinical presentation. * Cutoff forseropositivity was determined as the mean value (for each particular variable measured) plus 3 SD of the Swedish non-immune controls.

PfEMP1				
Peptide index	Peptide sequence	Peptide number	Protein ID	p-value
PEP_36115	LRWYDEWADDFC	305	PFIT_bin06900	0.041586
PEP_36136	VKDTCQGYNNSG	326	PFIT_bin06901	0.041586
PEP_36233	DTKFVATLKEAP	423	PFIT_bin06902	0.041586
PEP_36580	IEDNNKQISIQH	770	PFIT_bin06903	0.022392
PEP_36732	GENHNSNLQSSW	922	PFIT_bin06904	0.032227
PEP_36794	FLRWFNEWSEDF	984	PFIT_bin06905	0.032227
PEP_36877	YINVNGLEPYEF	1067	PFIT_bin06906	0.032227
PEP_37304	IEFLDKTNDTCH	1494	PFIT_bin06907	0.032227
PEP_37517	NGTDRKTGEDLW	1707	PFIT_bin06908	0.041586
PEP_37752	YHDNTNNWDSRG	1942	PFIT_bin06909	0.041586
PEP_37822	NNSRDGLNAMMF	2012	PFIT_bin06910	0.041586
PEP_37983	LNIKYQNIKASN	2173	PFIT_bin06911	0.041586
PEP_38044	CPNLEFPDDTFE	2234	PFIT_bin06912	0.041586
PEP_38045	PNLEFPDDTFEY	2235	PFIT_bin06913	0.032227
PEP_38223	SKSDGHIPHSAG	2413	PFIT_bin06914	0.041586
PEP_38288	LDDGMEEKPFIT	2478	PFIT_bin06915	0.041586
PEP_38320	TNSMDDPKYVSN	2510	PFIT_bin06916	0.041586
PEP_38324	DDPKYVSNNVYS	2514	PFIT_bin06917	0.041586
PEP_38479	ENPFVDDIPMDH	2669	PFIT_bin06918	0.041586

RIFIN-A				
Peptide index	Peptide sequence	Peptide number	Protein ID	p-value
PEP_74129	SLPLNILEHNPW	12	PFIT_bin05750	0.032227
PEP_150830	KKKMKKKLQYIK	315	PFIT_bin05750	0.022392
PEP_54144	KKMKKKLQYIKL	316	PFIT_bin05750	0.022392

$SURFIN_{4.2}$				
Peptide index	Peptide sequence	Peptide number	Protein ID	p-value
PEP_129877	IILNKEENSPTE	238	PFIT_0422600	0.041586
PEP_130232	TMELPNQQEVFG	593	PFIT_0422600	0.011719
PEP_130237	NQQEVFGLYSPV	598	PFIT_0422600	0.041586
PEP_130397	MFLVFNKMNPFG	758	PFIT_0422600	0.041586
PEP_140915	INGEIPLKKEEW	947	PFIT_0422600	0.041586
PEP_130680	EEWFYNLKNEWI	1039	PFIT_0422600	0.041586
PEP_130882	EKWFEDLNNEWN	1241	PFIT_0422600	0.004546
PEP_128755	IEQSDHMEDMLM	1401	PFIT_0422600	0.041586
PEP_128782	KYMLEKWNKEEW	1428	PFIT_0422600	0.032227
PEP_131128	MNEWKEEKWFDK	1600	PFIT_0422600	0.041586
PEP_131578	CKRYWKDLDKMW	2050	PFIT_0422600	0.041586

Table S4. Peptides correlated with rosette disruption capacity in children sera. A small subset of samples was selected and tested on a peptide array including three surface antigens. The samples analyzed were divided according to their ability to disrupt rosettes (of parasites grown in group O RBCs), with 7 being positive while 5 were considered negative. Each sub-table represents peptides identified for each surface antigen. Peptides are in green represent those preferentially recognized by samples with rosette-disrupting capacity (positive) while those in red were preferentially recognized by sample without rosette-disrupting capacity (negative).
